# Religiousness, Health, and Depression in Older Adults from a Brazilian Military Setting

**DOI:** 10.5402/2012/940747

**Published:** 2012-08-09

**Authors:** Giancarlo Lucchetti, Alessandra L. G. Lucchetti, Mario F. P. Peres, Alexander Moreira-Almeida, Harold G. Koenig

**Affiliations:** ^1^Research Department, São Paulo Medical Spiritist Association, 04520-000 São Paulo, SP, Brazil; ^2^Department of Geriatrics, São Paulo Air Force Hospital, 02012-021 São Paulo, SP, Brazil; ^3^Research Department, João Evangelista Hospital, 02340-000 São Paulo, SP, Brazil; ^4^Department of Neurology, Federal University of São Paulo, 04021-001 São Paulo, SP, Brazil; ^5^Department of Psychiatry, Federal University of Juiz de Fora, 36036-900 Juiz de Fora, MG, Brazil; ^6^Department of Psychiatry and Department of Medicine, Duke University Medical Center, Durham, NC 27710, USA

## Abstract

This study aims to analyze the association between religious attendance, self-reported religiousness, depression, and several health factors in 170 older adults from a Brazilian outpatient setting. A comprehensive assessment was conducted including sociodemographic characteristics, religious attendance, self-reported religiousness, functional status, depression, pain, hospitalization, and mental status. After adjusting for sociodemographics, (a) higher self-reported religiousness was associated with lower prevalence of smoking, less depressive symptoms, and less hospitalization and (b) higher religious attendance was only associated with less depressive symptoms. Religiousness seems to play a role in depression, smoking, and hospitalization in older adults from a Brazilian outpatient setting. Self-reported religiousness was associated with more health characteristics than religious attendance.

## 1. Introduction

Older adults have higher levels of religious involvement than younger adults [1, 2]. The World Values Survey evaluated 87 countries (256,000 persons) from all around the World and found that those aged 50 years or more attended religious services more often and also were more likely to indicate that they were religious and spiritual than those aged less than 50 years old.

In addition, religiousness seems to have an impact on older adults' health as well. According to several studies, religiousness and spirituality were associated with lower prevalence of depression [3], better quality of life [4], less cardiovascular events [5], and even greater survival [5, 6].

However, studies dealing with the impact of religiousness in older medical outpatients, particularly in countries outside North America and Europe, are not frequent in the medical literature.

In Brazil, there are approximately 16.2 million older adults; 95% of the population have a religious affiliation (Catholics and Protestant Evangelicals in majority) and 37% attend religious services at least once a week [2].

Previous studies in Brazil have found an association between religiousness, alcohol, and mental health in older groups [7, 8] but have not evaluated other health factors such as sleep problems and hospitalization.

We seek to analyze the association between religious attendance, self-reported religiousness, depression, and several health factors in older adults from a Brazilian outpatient setting.

## 2. Patients and Methods

### 2.1. Type of Study and Location

A cross-sectional study was conducted at the general medical outpatient clinic of the São Paulo Air Force Hospital, Brazil, between February 2007 and December 2007. The São Paulo Air Force Hospital (SPAFH), Brazil provides care for the Brazilian Air Force in the state of São Paulo (region in South Eastern Brazil with approximately 40 million inhabitants) and treats military personnel, retired military, and dependents of current and retired military. Data were extracted from a study regarding implementation of a comprehensive geriatric assessment (CGA) in general practice [9].

### 2.2. Subjects

All patients who consented and saw general practitioners during this period were included. A patient was eligible if he or she was aged 60 years or older, was cognitively well (capable of answering the questions) and physically well enough to undergo the evaluation. After fully explaining the procedures and obtaining written informed consent from the patient, a general practitioner conducted a CGA. The present study was approved by the Ethics Committee of São Paulo Air Force Hospital.

### 2.3. Procedures

Further details regarding the original study procedures and methodology can be found in a previous publication [9]. In summary, a CGA was performed by a general practitioner including:

- sociodemographic data (sex, age, race, marital status, and education);
- number of medical diagnosis and medications in use;
- smoking and drinking habits (prevalence and frequency);
- depression (using Geriatric Depression Scale-15 validated into Portuguese) [10]. GDS-15 is a 15-item inventory with a yes/no format. Depressive symptoms were screened using a cut-off value ≥5 to indicate clinically significant depressive symptoms;
- cognition (using mini mental state evaluation validated into Portuguese) [11]. MMSE is a 30-point questionnaire test that is used to screen for cognitive impairment. A cutoff point of 19/20 on the MMSE was used among those with no formal education and a cut-off of 23/24 for subjects who had attended school. Dementia diagnosis was determined following DSM-IV criteria;
- activities of daily living (using Katz Scale) [12]: The Index ranks adequacy of performance in the six functions of bathing, dressing, toileting, transferring, continence, and feeding. Clients are scored yes/no for independence in each of the six functions. A score of 6 indicates full function, 4 indicates moderate impairment, and 2 or less indicates severe functional impairment;
- self-reported sleep problems were evaluated through the question “Do you have sleep problems” with yes/no answers;
- hospitalization in previous 6 months (yes/no);
- physical activity (prevalence and frequency)—physical inactivity was considered in those exercising less than twice a week per 30 minutes;
- pain rating (using a visual analogic scale, graded from 0 to 10, in which zero means no pain and 10 very severe pain);
- social support was evaluated through the question “Do you have someone to count on when you're down?” with yes/no answer;
- religiousness was measured through religious attendance (extrinsic) question obtained from the Duke Religion Index validated into Portuguese [13, 14] divided into 6 answers: from “never attending” to “more than once a week” and a self-reported religiousness (intrinsic) question previously used in Duke NIMH Epidemiologic Catchment Area Study by Koenig et al. (1994) [15] asking about the importance of religion and faith to life with 3 possible answers: “not important”, “somewhat important,” and “very important.”

### 2.4. Data Analysis

Data analysis was performed using SPSS 17.0 for Windows. First, we conducted bivariate analyses (chi-square for categorical variables and *t*-test for continuous variables) testing associations between religiousness variables and health variables.

Logistic regression models were performed in order to assess the relation between religiousness (dichotomized) and health factors. All significant variables in bivariate analyses were included in multivariate models that controlled for gender, age, marital status, social support, functional status, and education.

Finally, a linear regression model (Table 4) was also performed in order to assess the relation between religious aspects (continuous) and GDS scores. The model was controlled as well for gender, age, marital status, social support, functional status, and education.

The level of significance was 0.05 and the confidence interval was 95%.

## 3. Results

One hundred and eighty seven patients attended to the clinic. From these, 17 were excluded: 8 because of incomplete physical or mental health data, 2 did not consent, and 7 patients were younger than the age of 60.

The final sample consisted of 170 elderly patients. There was a predominance of females (65.9%), with a mean age of 75.7 years old (range: 60 to 97) and 8.1 years of education. Most patients lived in the city of São Paulo, 54 (31.8%) were retired military personnel and 116 (68.2%) were dependents/spouses of retired or current military personnel.

In our sample, there was a predominance of females in their mid 70s, in accordance with the findings of other studies conducted in Brazil's elderly population [8, 16]. In comparison with the Brazilian general population, the study participants had a higher educational and socioeconomic level, and the majority was Caucasian [9].

Subjects had an average 9.07 (±3.24) clinical diagnoses, 30 (17.6%) were current smokers and 12 (7.1%) were alcoholics. Depression was found in 56 (32.9%) patients and cognitive impairment in 33 (19.4%) patients.  Table 1 shows patients' characteristics.

Concerning religious aspects, 98 (60.1%) were Roman Catholics, 23 (14.1%) Evangelical Protestants, 19 (11.7%) Spiritists, and only 4 (2.5%) did not indicate a religious affiliation. These results are in line with the last national survey in which the most frequent affiliations were Catholicism (68%) and Protestant/Evangelicals (23%). Nevertheless, in our study we found a high percentage of Spiritists (11.7%) compared to the Brazilian population (2.5% were Spiritists) [2]. When questioned about religious aspects, 75 (46.0%) declared attending religious services once per week or more and 103 (60.3%) of patients stated religion and faith were “very important”.

Bivariate analysis can be found in Table 2. The following variables showed statistically significant associations with higher religious attendance (once a week or more): lower prevalence of depression and less alcohol use. Higher self-reported religiousness was associated with less depression, less hospitalization in previous 6 months, and less smoking.

After adjusting for sociodemographic aspects (Table 3), (a) higher self-reported religiousness was associated with a lower prevalence of smoking (OR: 0.28 (CI 95%: 0.10–0.77)), less depression (OR: 0.19 (0.08–0.44)), and less hospitalization in previous 6 months (OR: 0.34 (0.13–0.85)); (b) higher religious attendance was only associated with less depression (OR: 0.28 (0.12–0.64)).

If we consider lower religiousness as the reference, we will find that: (a) lower self-reported religiousness was associated with a higher prevalence of smoking (OR: 3.47 (1.29–9.36]), higher prevalence of depression (OR: 5.13 (2.25–11.68)) and more hospitalization in previous 6 months (OR: 2.89 (1.16–7.15)); (b) low religious attendance was only associated with a higher prevalence of depression (OR: 3.48 (1.55–7.85)).

In a separate group analysis, Roman Catholics with higher religious attendance (OR: 0.26 (0.08–0.78), *P* = 0.016) and higher self-reported religiousness (OR: 0.17 (0.06–0.53), *P* = 0.002) presented less depression.

Less depressive symptoms (lower GDS scores) were also associated with higher religious attendance (Beta: −1.045, *P* < 0.001) and higher self-reported religiousness (Beta:−1.010, *P* < 0.001), even after adjusting for sociodemographic aspects (Table 4).

## 4. Discussion

Religious and spiritual beliefs are important aspects for older Brazilian adults' lives that may have positive or negative effects on health. In our sample, we found a predominance of Roman Catholics (almost two-thirds), followed by Evangelical Protestants and spiritists, which is consistent with previous Brazilian studies [2, 17]. Nevertheless, this profile is different from US studies in which there is a Protestant majority [18]. This underscores the importance of replicating these findings in other sociocultural backgrounds, such as South American countries. Brazil has a unique background, being the most populous Catholic country in the world with approximately 125 millions of Roman Catholics [2] and is also the country with the highest number of followers of Kardec's Spiritism in the world [19].

Bivariate analysis showed that lower religious attendance was associated with greater depression and alcohol use. These results persisted only for depression in the logistic regression model.

Self-reported religiousness, however, was associated with less depression, greater physical activity, less hospitalization in the previous 6 months, and less smoking. After controlling, other variables such as depression, hospitalization in previous 6 months, and smoking remained significant.

Religiousness was not associated with dementia, presence of pain, and sleep problems.

The relationship between spirituality/religiousness and depression is well known in the literature. Studies have shown that higher spirituality/religiousness levels are associated with less depression [7, 8, 20], similar to our study. Furthermore, studies have also shown faster remission of depression and greater well-being in those with higher religiousness [21]. The exact cause for that is not totally understood.

The findings from the present study are consistent with those from another Brazilian study [22], in which higher religiousness was associated with lower prevalence of depression in hospitalized patients. In our case, depression was the only factor that was inversely related with both religious dimensions (religious attendance and self-reported religiousness), supporting the importance of religiousness in perhaps preventing depression.

Another important health outcome was hospitalization in the previous 6 months. Some international studies—prospective [15] and retrospective [15, 23]—have found an inverse relationship between hospitalization days and spirituality/religiousness. It has been hypothesized [15] that religion may decrease health service use by reducing actual need (promoting better physical and mental health), reducing perceived need (lowering overuse or unnecessary use of services), decreasing perceived benefit (providing alternative methods for healing and reducing manipulation of health care system for other purposes), increasing timely access to and availability of health services (increasing monitoring for health problems and encouraging better compliance), and increasing motivation to seek care (by reducing depression and other negative mental health states that interfere with diagnosis and treatment).

In the present study, lower tobacco use was also associated with higher levels of religiousness, similar to the findings of other studies [7, 24, 25]. In 2008, Ward et al. [24] carried out a study with young adults that found an inverse relationship between tobacco use and religiousness, as did Benjamins et al. in Mexican elderly [25]. Concerning Brazil, we found only one study dealing with this subject, which indicated that elderly who do not have a religion have a 124% increased risk of smoking [7]. Some authors believe that religious participation can increase coping skills, instill moral values, and reduce the likelihood of turning to alcohol, smoking, or other drugs during times of stress [15]. Unlike other studies [7], alcohol consumption was not associated with religiousness in our population; we believe that this was due to the small number of alcohol users in the present sample.

Our findings obtained from this military setting are in line with other military international studies. McLaughlin et al. [26] evaluated outpatients at a military medical center and found that compared to the general US population, a higher percentage of military outpatients attends religious services once a week or more, which was also found in our study. Fontana and Rosenheck [27] also evaluated military veterans and found that religious faith was an important predictor of mental health services use.

Another interesting finding is the difference between religious attendance and self-reported religion. Almost 45% of the patients attended religious services at least once a week, similar to other studies [2]. However, more than 60% stated that religion was very important in their lives. In addition, self-reported religion was associated with more health characteristics than was attendance at religious services. These results suggest that older persons, possibly due to difficulties in walking, transportation, and disability, may not be able to attend religious services. Nevertheless, they still cultivate a more private, subjective religiousness, and the latter can still impact their lives.

In other words, attendance is more related to an extrinsic religiousness whereas self-reported religiousness is more related to intrinsic religiousness. According to Allport's work [28], an intrinsic religious orientation is described as being more mature in that the believer views religion as an end in itself, that is, the believer believes without clearly identifiable external motives for doing so. In contrast, an extrinsic religious orientation is immature and is more of a means to some other end, that is, belief is motivated by external factors (social acceptance and advancement), which is also more correlated with prejudice. Older persons seem to give more attention to transcendent issues in contrast to social acceptance and that, in part, could explain our findings.

Study limitations must be considered while evaluating these results. First, the study is cross-sectional, not allowing cause-effect conclusions. Second, different from other Brazilian studies, this sample had a mean of 8.1 years of education, higher than other studies conducted in Brazil. This is probably explained by the setting, a military hospital with higher educated patients. Third, the study has examined a simplistic construct of religiousness (using self-reported religiousness and religious attendance). Forth, the study analysis evaluated a fairly high number of comparisons. Nevertheless, when a more stringent level of significance would have been chosen (*P* < 0.010, e.g.), the findings would be largely identical, suggesting that the study did not yield spurious associations.

Nevertheless, the present study has some strengths that should be highlighted: (a) the replication of a research on religiousness and depression in a different cultural setting than most of the research available, (b) the adjustment for social support, and (c) the adjustment for physical health status.

Finally, we believe that studies conducted in different cultures, such as South American countries, are necessary to understand the impact of religiousness and spirituality in older adults. Similar to other international studies, religiousness may have a protective effect against depression, smoking, and hospitalization in older adults from this Brazilian outpatient setting.

## Figures and Tables

**Table 1:** Sociodemographic, health, and religious variables of the study sample.

Patients' baseline characteristics	
Age (±SD)	75.75 (±8.06) years
Gender	
Female	112 (65.9%)
Male	58 (34.1%)
Number of medical diseases	9.07 (±3.24)
Years of education (±SD)	8.1 (±2.12) years
Activities of daily living	
Totally dependent	13 (7.6%)
Partially dependent	5 (2.9%)
Independent	152 (89.4%)
Alcohol use	
Occasionally	36 (21.2%)
Alcoholic	12 (7.1%)
Dementia	33 (19.4%)
Depression	56 (32.9%)
Hospitalization in previous 6 months	33 (19.4%)
Physical activity	67 (39.4%)
Presence of Pain	70 (41.7%)
Self-reported sleep problems	90 (52.9%)
Smoking	30 (17.6%)
Religious attendance	
Never	75 (46%)
Less than once a week	15 (9.2%)
Once a week or more	73 (44.8%)
Importance of religion in life	
Not important	33 (20.2%)
Somewhat important	27 (16.6%)
Very important	103 (60.3%)

SD: standard deviation.

**Table 2:** Factors associated with religiousness using bivariate analysis, Air Force Hospital of São Paulo-Brazil, 2007.

Health variables	Religious attendance				Importance of religion to life			
	Less than once a week	Once a week or more	*P*	Odds-ratio (95% CI)	Not or somewhat important	Very important	*P*	Odds-ratio (95% CI)
Any alcohol use	35.6%	20.5%	0.035^∗^	2.13 (1.04–4.35)	36.7%	24.3%	0.092	1.80 (0.90–3.60)
Dementia	20.0%	11.0%	0.110	2.03 (0.82–4.98)	23.3%	11.7%	0.054	2.30 (0.98–5.39)
Presence of depression	41.1%	21.9%	0.009^∗^	2.48 (1.24–4.98)	48.3%	23.3%	0.001^∗^	3.07 (1.55–6.09)
Hospitalization in previous 6 months	21.1%	13.7%	0.213	1.68 (0.72–3.89)	30.0%	10.7%	0.001^∗^	3.58 (1.55–8.25)
Physical activity	36.7%	46.6%	0.203	0.66 (0.35–1.24)	31.7%	46.6%	0.061	0.53 (0.27–1.03)
Pain	43.3%	39.7%	0.641	1.16 (0.61–2.17)	41.7%	41.7%	0.991	0.99 (0.52–1.99)
Self-reported sleep problems	55.6%	45.2%	0.185	1.51 (0.81–2.81)	51.7%	50.5%	0.882	1.04 (0.55–1.98)
Smoking	20.0%	12.3%	0.193	1.77 (0.74–4.23)	26.7%	10.7%	0.008^∗^	3.04 (1.30–7.09)

^∗^Statistically significant chi-square test (*P* < 0.05).

**Table 3:** Health factors associated with higher religiousness (logistic regression)^#^.

	Presence of depression	Hospitalization in previous 6 months	No consumption of alcohol	Smoking
	Adjusted OR (95% CI)	Adjusted OR (95% CI)	Adjusted OR (95% CI)	Adjusted OR (95% CI)
Religious Attendance				
Once a week or more	0.28 (0.12–0.64)^∗^ 1.00	1.04 (0.39–2.78) 1.00	1.29 (0.19–8.45) 1.00	0.41 (0.14–1.17) 1.00
Less than once a week	Wald: 9.118, *P* = 0.003	Wald: 0.007, *P* = 0.935	Wald: 0.071, *P* = 0.790	Wald: 2.772, *P* = 0.096
Importance of religion to life				
Very important	0.19 (0.08–0.44)^∗^ 1.00	0.34 (0.13–0.85)^∗^ 1.00	1.02 (0.23–4.49) 1.00	0.28 (0.10–0.77)^∗^ 1.00
Not or somewhat important	Wald: 15.210, *P* ≤ 0.001	Wald: 5.273, *P* = 0.022	Wald: 0.001, *P* = 0.976	Wald: 6.073, *P* = 0.014

^#^All significant variables in the bivariate model were included for multivariate logistic model, controlled by gender, age, marital status, social support, functional status, and education.

^∗^Statistically significant.

**Table 4:** GDS scores and religious aspects (linear regression)^#^.

	**β**	Std. error	Beta standardized	*P*
Religious attendance	−1.045	0.225	−0.339	<0.001^∗^
Importance of religion to life	−1.010	0.306	−0.271	<0.001^∗^

^#^The relation between GDS scores and religious aspects was also included in a linear regression, controlled by gender, age, marital status, social support, functional status, and education.

^∗^Statistically significant.
